# Women in gender equality movement: a systematic literature review

**DOI:** 10.3389/fsoc.2024.1432383

**Published:** 2024-12-16

**Authors:** Syifa Siti Aulia, Marzuki, Suyato, Iqbal Arpannudin

**Affiliations:** Civic Education Study Program, Universitas Negeri Yogyakarta, Yogyakarta, Indonesia

**Keywords:** leadership, women, movement, social, equality, gender

## Abstract

This study employs a rigorous Systematic Literature Review (SLR) methodology to examine the role of women’s leadership in advancing social movements for gender equality. Utilizing Scopus-indexed articles, we provide a comprehensive review of the relevant literature, analyzing factors contributing to the success of women leaders, effective leadership strategies, and the impacts of these efforts on communities and structural change. The findings indicate that women’s leadership plays a crucial role in fostering sustainable social change by enhancing women’s participation in decision-making, increasing public awareness of gender issues, and advocating for policies that promote gender equity. Additionally, this review identifies key challenges faced by women leaders in social movements, including gender stereotypes, institutional barriers, and inequities in resource access. In conclusion, strengthening women’s leadership in social movements is essential for advancing gender equality, though significant systemic obstacles must still be addressed to fully realize the transformative potential of such leadership.

## Introduction

The start of the Education for All (EFA) movement opened a new era in education with a focus on gender equality and women’s empowerment (Holmarsdottir et al., 2011). The United Nations “HeForShe” movement highlighted efforts to elevate thoughts and ideas for gender equality as a human rights issue in which everyone can participate (Stowers et al., 2019). The journey towards gender equality is evolving gradually, with qualitative research conducted on 20 couples in Singapore revealing that power in couple relationships is beginning to be equalized (Quek and Knudson-Martin, 2008). Data from survey results on women and men in 51 countries show that women’s activism affects gender equality in various countries and is the most successful organization in promoting equality (Akchurin and Lee, 2013). Research in China in 2018 involving 862 respondents suggested the need to improve education on gender equality, raise awareness of relevant laws and regulations, and create an organizational culture and environment that supports gender equality (Wang et al., 2022).

In other countries in the world, gender equality problems are still urgent to address. Gender inequality is a major problem in most developing countries. The 2011–2012 European research project, “The Role of Men in Gender Equality,” revealed that masculinity is being increasingly applied in practice as women’s education and professional roles increase (Scambor et al., 2014). Patriarchal norms limit women’s participation in various areas of life, including education, health, and the economy, which damage the economic development of those countries (Pervaiz et al., 2023). Studies show that gender equality is substantial for economic development (Klasen and Lamanna, 2009; Orfan and Samady, 2023; Pervaiz et al., 2023). Investing in gender equality may assist economic growth and development in developing countries (Pervaiz et al., 2023).

Negotiations for gender equality and women’s empowerment are considered to challenge social practices that have prioritized the interests of men (Kirton, 2021). Gender issues in social, political, and economic life are among the elements that need attention. Gender inequality, which has always been a significant challenge in building an inclusive and fair democracy, is a fundamental problem in developing countries such as Indonesia. In the Indonesian context, there are still obstacles or difficulties in achieving gender equality due to the culture that still values women staying at home rather than working (Audina, 2022). A study conducted by Cameron (2023) observed that Indonesia has more gender inequality than other neighboring countries but has less gender inequality than most Muslim countries in the world, regardless of income level. However, economically, women’s participation in Indonesia is lower than its growth rate. Low female labor force participation is caused by women quitting their jobs after marriage and having children, especially in the formal sector, as firms in the formal sector do not provide flexible working conditions to improve their ability to retain female employees.

Various problems regarding equality in Indonesia include, among others, the implementation of gender equality in Indonesia not being realized (Sari and Ismail, 2021). Gender equality has not yet reached the peak of the struggle; people are still arguing about the role of women (Suhada, 2021). The condition of gender discrimination in Indonesia still occurs in all aspects of life and tends to improve (Arifin, 2020). The National Education System Law, the Human Rights Law, and other sharing regulations guarantee equal opportunities for men and women to acquire knowledge.

Gender equality refers to equality in rights, opportunities, and individual treatment despite their sex (Elder-Vass, 2017; PCAR, 2013). Ideally, men and women should have the same access to resources, education, health services, job, and decision-making power. Women’s empowerment, on the other hand, particularly focuses on empowering women and girls to create an environment where they can completely use their rights and have control over their own lives. It involves providing equipment, knowledge, and support to women in challenging the gender norms and stereotypes, and to actively participating in social, economic, and politic fields. Both the token theory and critical mass theory suggest that achieving gender balance is not considered essential, and that this belief contributes to the current situation. Additionally, women utilize their roles within traditional family structures to pursue their political ambitions despite existing patriarchal norms (Patel et al., 2023).

To understand the importance of gender equality and women’s empowerment comprehensively, it is important to define the following terms. Gender equality refers to equal rights, responsibilities, and opportunities for every individual of any gender, while women’s empowerment focuses on developing and promoting women’s rights and agencies. Community perspectives and power play an important role in creating one’s identity and gender.

Thus, this study aims to show the urgency of women’s leadership and social movement through a systematic literature review of concepts and methods. The benefits of this research include (1) Benefits for the development of gender studies on civic education practices in higher education through enriching the reference of best practices in gender studies that have evolved in many countries; (2) Benefits for policymakers: understanding the urgency of the existence of gender and woman leadership at social movement; and (3) Benefits for future researchers: identifying the research gap for further development of research in the field of gender empowerment studies.

## Method

This research employs a Systematic Literature Review (SLR) approach, a structured method commonly used to collect, analyze, and synthesize existing studies on a specified topic. The SLR methodology facilitates a comprehensive examination of the literature by following rigorous selection and analysis protocols, ensuring that the findings are reliable and generalizable. This study aims to address the research question: “Does social movement led by women have a greater impact on achieving gender equality?”

### PICOS framework

To enhance methodological rigor in identifying and analyzing relevant studies, the PICOS Framework (Population, Intervention, Comparison, Outcome, Study Design) was utilized. The PICOS framework is essential for systematically selecting and analyzing literature that aligns with the research objectives, allowing for a structured and replicable review process. Each component of the PICOS framework is tailored to this study as follows:

- Population: The focus is on women engaged in social, economic, or political contexts pertinent to gender equality movements. This includes women participating in activism, leadership, or advocacy roles within movements that promote gender equality.
- Intervention: This element covers the actions or policies implemented to improve women’s conditions, such as educational programs, public policy initiatives, or advocacy campaigns aimed at advancing gender equality. These interventions represent specific efforts within women-led social movements to address gender disparities.
- Comparison: The comparison involves evaluating the impact of these interventions by contrasting them with alternative or control conditions. For example, the effects of women-led initiatives may be compared to those of mixed-gender or male-led movements, allowing an assessment of the unique contributions of female leadership in promoting gender equality.
- Outcome: Outcomes are drawn from previous studies and may include indicators of gender equality such as increased political participation of women, improved access to education and employment opportunities, and shifts in societal attitudes toward gender roles. This component synthesizes findings on tangible benefits stemming from women-led movements.
- Study Design: Studies are categorized based on their research design, which may include qualitative, quantitative, or action research methodologies. This categorization allows for an in-depth examination of how different methodological approaches contribute to understanding women’s roles and impact in gender equality movements.

## Data collection and search strategy

The primary data source for this research is peer-reviewed articles published in Scopus-indexed journals, ensuring a high standard of academic credibility. The initial literature search was conducted using the Scopus search engine with a predefined search string:

TITLE-ABS-KEY((“social movement” OR “social activism” OR “activism”) AND (“female leadership” OR “women leadership” OR “female-led” OR “women-led”) AND (“gender equality” OR “gender equity” OR “gender parity”)).

This query generated an initial pool of 829 articles. These articles were screened based on established inclusion and exclusion criteria to ensure relevance to the study focus. Articles published between 2014 and 2024, written in English, and appearing in Scopus-indexed journals were included. Additionally, studies not directly related to gender equality (e.g., those focusing solely on Environmental Science, Medicine, or Engineering) were excluded from further analysis. This screening process narrowed the selection to 99 articles that met all criteria for relevance and quality (see Table 1 for screening details).

**Table 1:** Screening criteria summary.

Screening criteria	Description	Number of documents
Search string	TITLE-ABS-KEY((“social movement” OR “social activism” OR “activism”) AND (“female leadership” OR “women leadership” OR “female-led” OR “women-led”) AND (“gender equality” OR “gender equity” OR “gender parity”))	829
Year	2014 to 2024	688
Subject area	Does not include articles with the subjects Environmental Science; Energy; Agricultural and Biological Sciences; Medicine; Engineering; Computer Science; Earth and Planetary Sciences; Health Professions; Decision Sciences; Nursing; Multidisciplinary; Mathematics; Biochemistry, Genetics and Molecular Biology; Business, Management and Accounting; Economics, Econometrics and Finance	440
Document type	Article	231
Source title	Not selected	
Publication stage	Finals	215
Keywords	Unrestricted, exclusion criteria are filtered through objective screening	
Affiliation	Not selected	
Funding sponsor	Not selected	
Country/territory	Not selected	
Source type	Journal	215
Language	English	209
Open access	All open access	99
Total	99 journal articles	

## PRISMA guidelines

The article selection process was conducted following the PRISMA (Preferred Reporting Items for Systematic Reviews and Meta-Analyses) standards (Page et al., 2021), a rigorous guideline that ensures transparency and reproducibility in systematic reviews. PRISMA guidelines facilitate clear reporting of the study selection process, enhancing the review’s reliability by documenting each stage of filtering and selection.

This systematic approach enables a robust synthesis of existing literature, allowing for an in-depth exploration of the impact of women-led social movements on gender equality outcomes. By focusing on interventions led by women, the study contributes to a nuanced understanding of gender equality efforts globally and locally, providing valuable insights into the unique role of female leadership in social activism.

This targeted selection process, guided by PRISMA standards (Page et al., 2021), ensured that only relevant studies were included, allowing for a focused exploration of women-led social movements’ impact on gender equality. By applying these systematic methods, the study achieves a robust literature synthesis, contributing to a deeper understanding of women’s contributions to gender equality efforts globally and locally.

Table 1 shows that the articles used in this research were published from 2014 to 2024. Only articles written in English in Scopus-indexed journals were selected. With this screening criteria, only 99 articles were appropriate to process further with the PRISMA diagram.

The exclusion applies to articles focusing on a broad range of topics including Environmental Science, Energy, Agricultural and Biological Sciences, Medicine, Engineering, Computer Science, Earth and Planetary Sciences, Health Professions, Decision Sciences, Nursing, Multidisciplinary, Mathematics, Biochemistry, Genetics and Molecular Biology, Business, Management and Accounting, Economics, Econometrics and Finance. This approach ensures a targeted exploration and discussion of specific subjects outside these extensive fields, enabling a more focused and specialized examination of other areas of interest.

## Results and discussion

### Results

This systematic review was written following careful and precise protocols to keep the accountability, integrity, and transparency of the report. PRISMA flow diagram contained a checklist to examine the report protocol comprehensiveness and help researchers follow the guidelines. PRISMA flow diagram could help reduce deviations in the selection and conclusion-drawing process. The information management process comprised selecting and including various collected documents.

**Figure 1:**
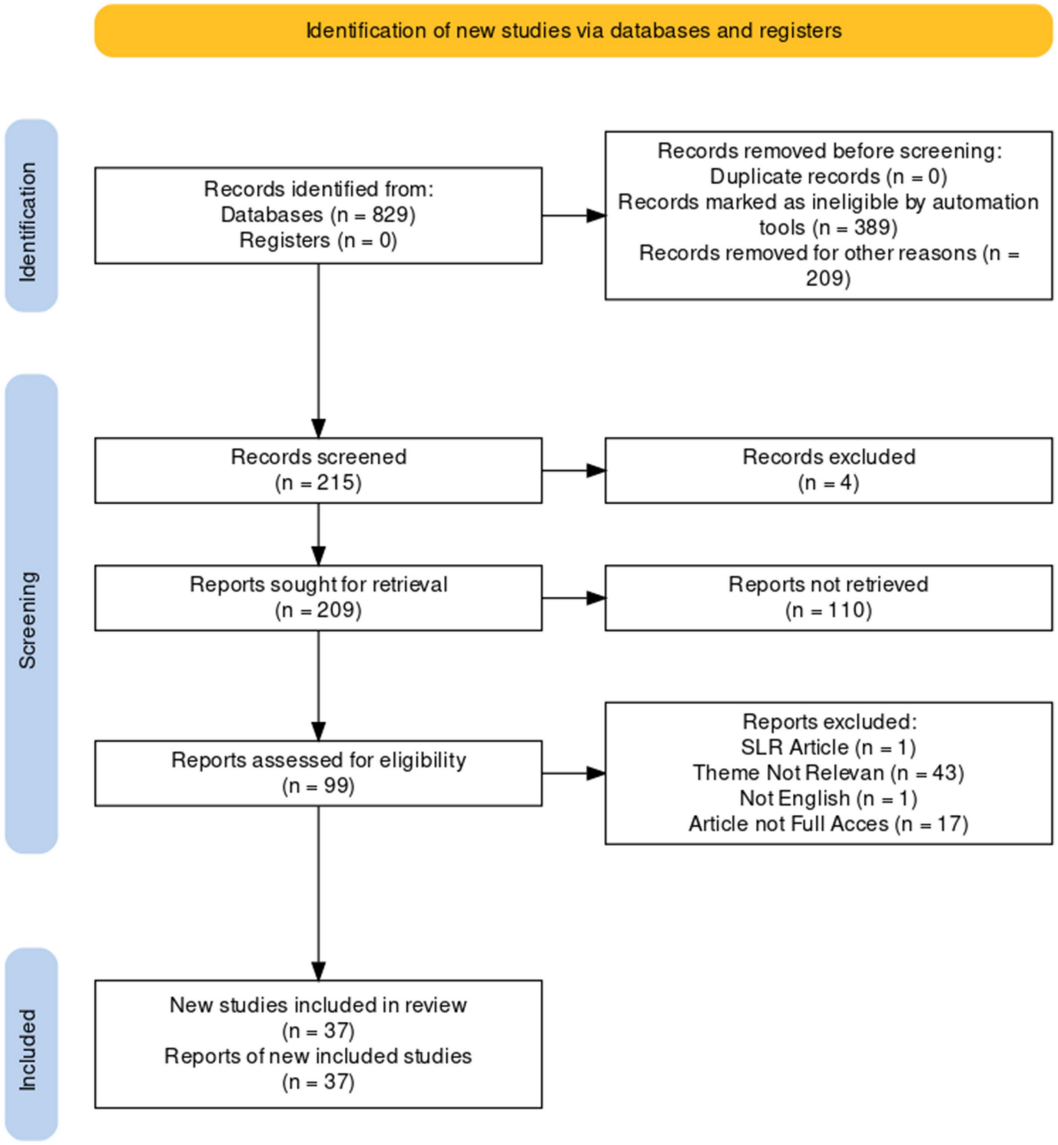
PRISMA model.

The subsequent selection of data sources followed the PRISMA stages and standards for systematic literature reviews, based on a multi-stage procedure consisting of three sequential stages, as follows: (a) Identification, (b) Screening, and (c) Included. The results of the second stage selection can be observed in Figure 1.

From the above PRISMA, 37 articles fitted the criteria, and the data extraction was carried out with the PICOS framework. The elimination of 52 articles stood for several reasons, such as one article being SLR, 43 articles being irrelevant to the topic, one article not written in English, and 17 articles not being accessed/not providing full access.

Researchers utilized Vos Viewer to analyze the 37 selected articles further to observe the trends by referring to the words in relevant titles and abstracts. Table 2 shows terms that appear frequently.

**Table 2:** Occurrences of terms.

Term	Occurrences	Relevance score	Term	Occurrences	Relevance score
Equality	9	1.8	Argentina	2	0.6
Career	5	12,217.0	Attitude	2	11,396.0
Right	5	3.8	Australia	2	12,001.0
Community	4	5.7	Bolivia	2	11,214.0
Ideology	4	12,562.0	Chile	2	5.2
State	4	4.6	Constitution	2	0.5
Struggle	4	12,156.0	Equal opportunity	2	13,967.0
Elite	3	4.3	Exclusion	2	5.2
Feminist	3	13,511.0	Family	2	5.7
Governance	3	4.7	Female	2	36,163.0
Highlight	3	3.1	Female body	2	5.2
Intersectionality	3	10,348.0	Feminism	2	13,511.0
Latin America	3	10,241.0	Global south	2	6.7
Movement	3	11,147.0	Mexico	2	11,214.0
Observation	3	5.8	National identity	2	4.3
Pursuit	3	3.4	Nationalism	2	12,039.0
Significance	3	2.7	Negative association	2	6.2
Status	3	0.3	Nigeria	2	12,364.0
Stereotype	3	14,172.0	Political party	2	26,291.0
Violence	3	6.0	Political subject	2	12,497.0
Women	3	5.0	Public sphere	2	6.9
Women’s experience	3	3.2	Thailand	2	3.5
Women’s leadership	3	6.0	Women’s right	2	5.0
Workplace	3	21,315.0			
Ability	2	4.1			

Source: VOSViewer (2024).

**Figure 2:**
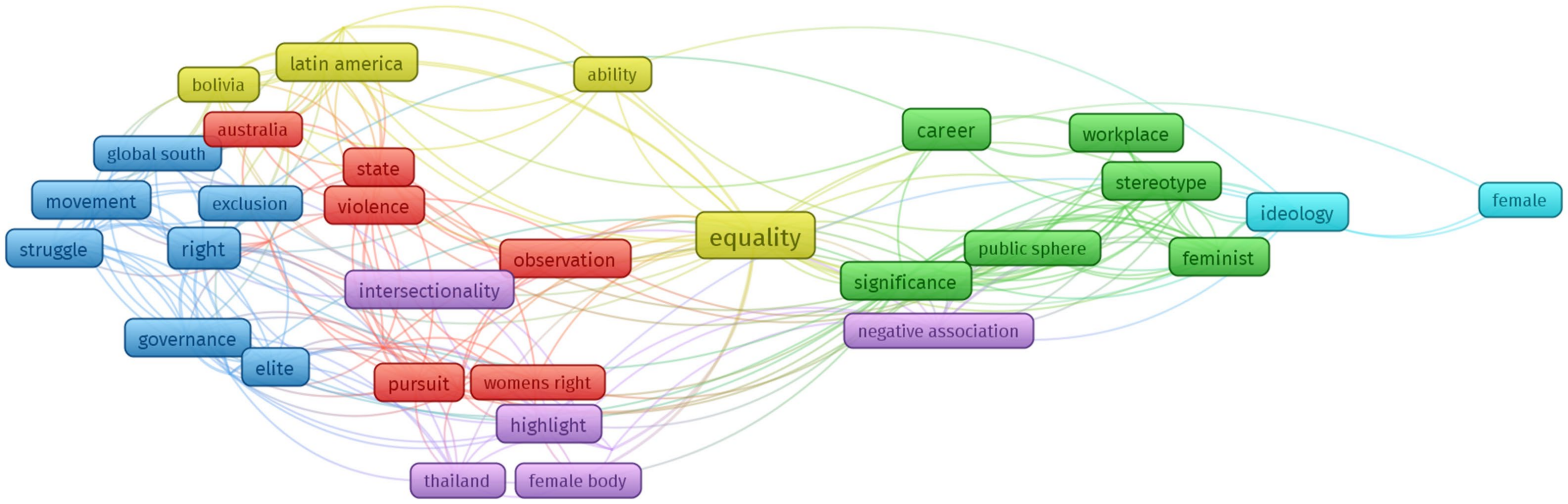
Network visualization.

From the result of the occurring terms selection are 48 words interrelated to each other with differing occurrence rates, including the terms *equality*, *career*, *right*, *community*, *ideology*, *state*, and *struggle*. Vos viewer visualization result of 37 articles generated six clusters as illustrated by the colors in the network visualization in Figure 2.

Figure 2 shows six research clusters denoted by different colors: red, green, blue, yellow, purple, and light blue. It shows the number of intersections and is considered close by the application. For example, in the red cluster, one has some intersecting terms.

**Figure 3:**
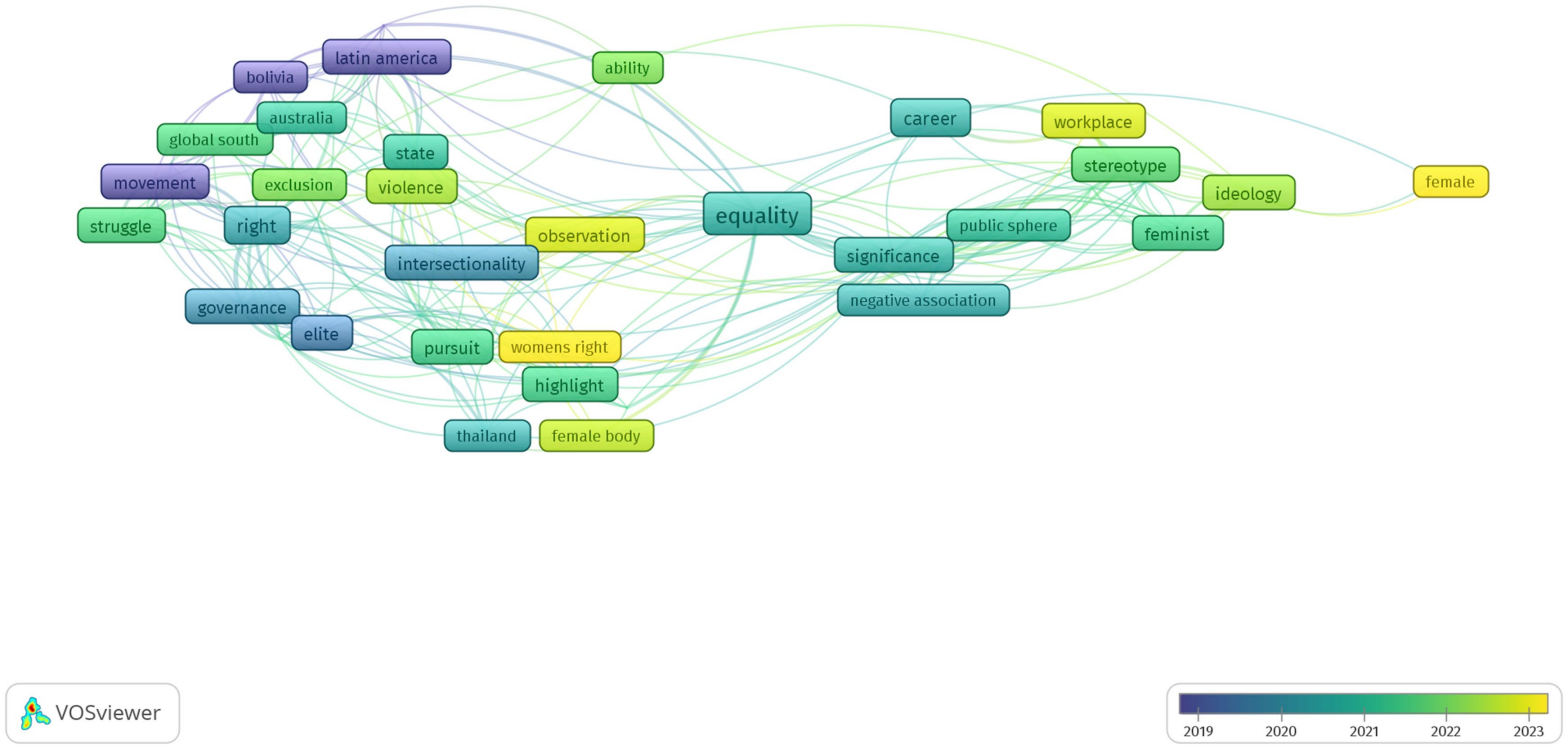
Overlay visualization.

On the other hand, the occurring terms based on overlay visualization can be observed in Figure 3.

The overlay depicts the average, and bright colors denote trends of the most recent years of the 37 articles analyzed. *Women’s rights, gender, workplace, ideology,* and *violence* are observed to be of interest to the application.

Many occurring terms are related to women’s leadership and movement as the themes are interrelated. It is observed that equality is related to *career*, *negative association, ability, workplace*, and those that show research trends on gender.

**Figure 4:**
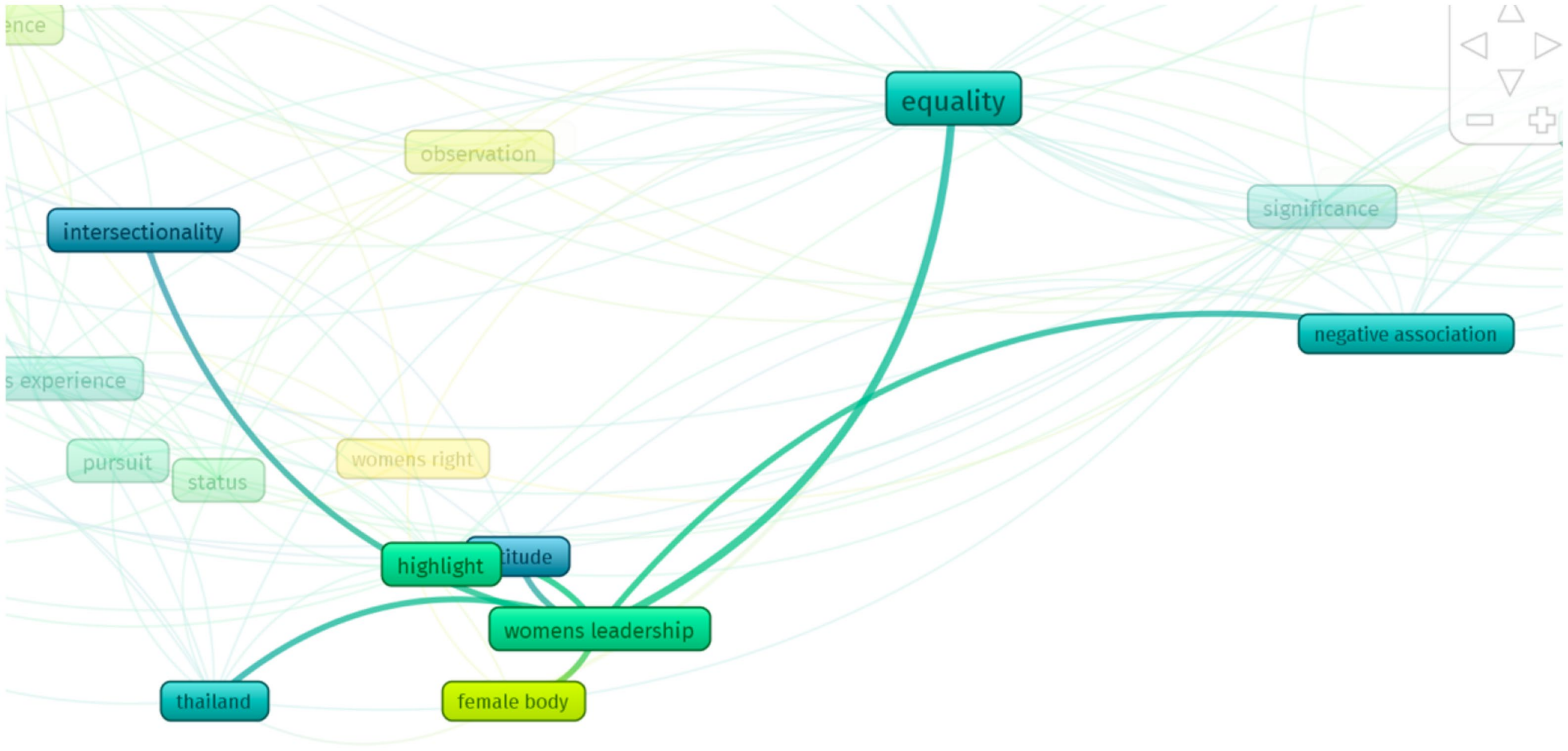
Women’s leadership terms.

The term *women leadership* is related to other terms in Figure 4.

The concept of female leadership is associated with negative stereotypes, intersectionality, attitudes, and the female body. Gender equality, a fundamental principle of human rights, involves eradicating all forms of discrimination against women. Conversely, women’s empowerment entails ensuring equal opportunities for women in education, healthcare, and the economy. Despite considerable efforts by many countries to achieve equality, it is apparent that women’s empowerment, as confirmed by Aziz et al. (2011) is not just essential, but a fundamental requirement for promoting women’s rights and development.

Gender equality encompasses the equal rights, opportunities, and fair treatment of individuals irrespective of their gender (Elder-Vass, 2017; PCAR, 2013). Research by Blanton et al. (2023) has shown that women’s empowerment, particularly in terms of their involvement in formal political processes and their civil liberties, results in active participation in civil society. In an ideal scenario, both men and women should have equal access to resources, education, healthcare services, and decision-making authority. Women’s empowerment, however, is not just an aspiration; it is a potent instrument for creating an environment in which women and girls can fully exercise their rights and take charge of their lives. This involves equipping them with the necessary tools, knowledge, and support.

It is important to remember the following: Women’s empowerment emphasizes promoting women’s agency and autonomy across various aspects of life (Fielding-Miller et al., 2020). Both gender equality and women’s empowerment are critical for creating a fair and inclusive community. Addressing gender inequality is essential for achieving justice and integrity, while also unlocking the full potential of individuals and the community for social and economic development (Cameron, 2023; Davies, 2018; Mitander, 2024).

To fully understand the significance of women’s empowerment and gender equality, it is important to define the following terms: Gender equality refers to equal rights, responsibilities, and opportunities for individuals of all genders, while women’s empowerment focuses on developing and promoting women’s rights and agency in particular. Public perspectives and power play a significant role in shaping one’s gender identity. Individuals often challenge gender norms established by society, and their experiences can influence their perception of themselves as men or women. In existentialism, a person’s biological characteristics determine how they identify themselves as a man or a woman. However, Foucault’s theory, Berger’s observation theory, and Mulvey’s Male Gaze theory highlight the influence of public perspectives and power in shaping one’s gender identity (Foucault, 1977; Kent, 2016; Mulvey, 1975).

**Figure 5:**
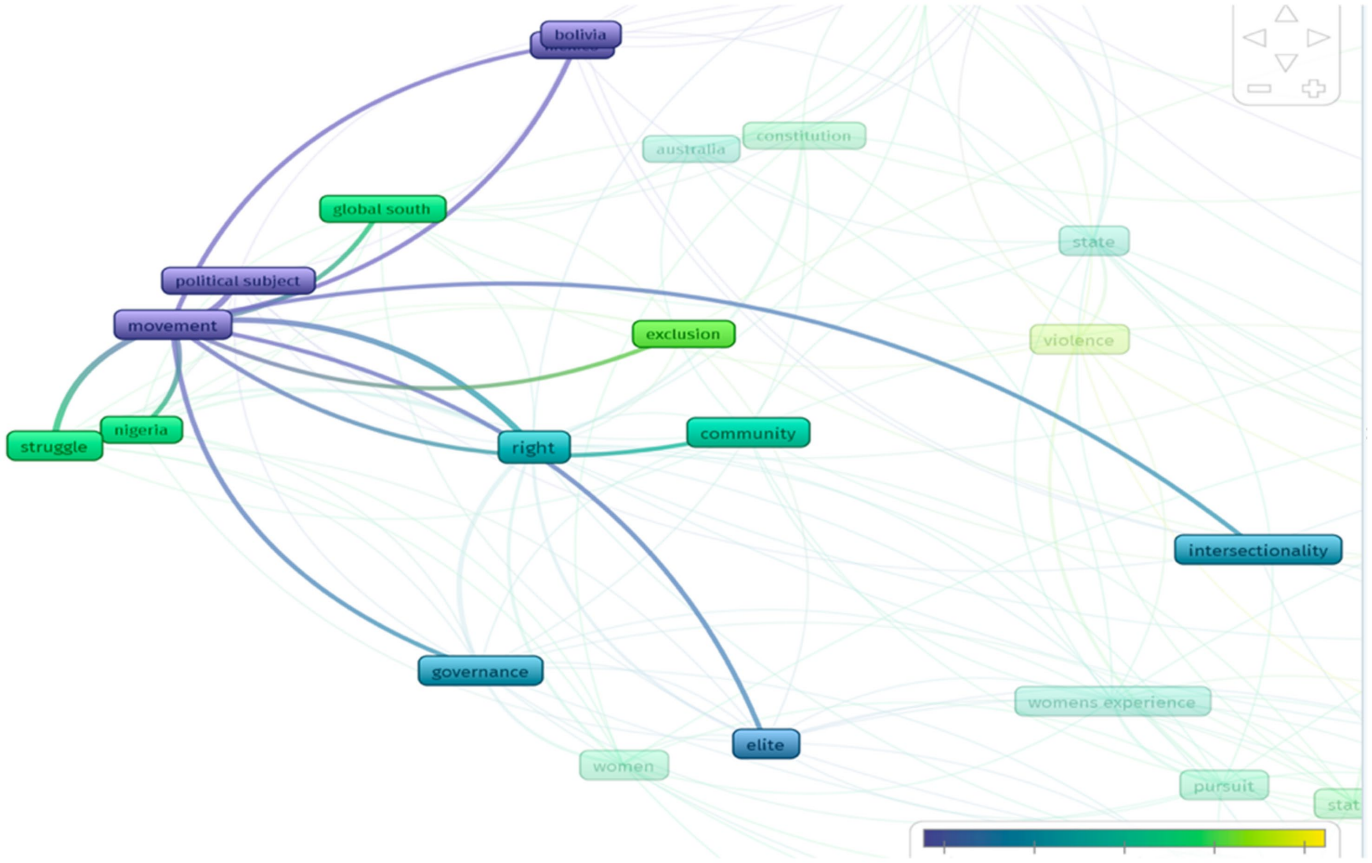
Term movement. Source: Vos Viewer (2024).

When highlighting the term *movement,* other related terms appear, such as *governance*, *elite*, *struggle*, *Nigeria*, *political subject*, *Bolivia*, *community*, and *global south*. The visualization is in Figure 5.

Women empowerment also places a particular emphasis on promoting women’s agency and autonomy in various life aspects (Fielding-Miller et al., 2020). Both gender equality and women’s empowerment are crucial in creating a fair and inclusive community. Resolving gender inequality is parallel with ensuring justice and integrity yet unlocking the full potential of both individuals and the community for social and economic development (Cameron, 2023; Davies, 2018; Mitander, 2024).

The research findings regarding the influence of women’s leadership on social movements strongly support the work of Evans et al. (2018) demonstrating a positive correlation between women’s empowerment and development across various sectors. The impact of women’s leadership on social movements is not only diverse, but also represents a rich tapestry of influence. This is clearly illustrated in the adaptation of the Women’s Empowerment in Agriculture Index (WEAI) by Pro-WEFI, which identifies areas of gender powerlessness relevant for prioritizing projects within the context of social movements (Rahman et al., 2024). Therefore, women’s leadership not only drives social change and the adoption of more inclusive policies but also has tangible, positive effects on promoting gender equality and sustainable development.

**Figure 6:**
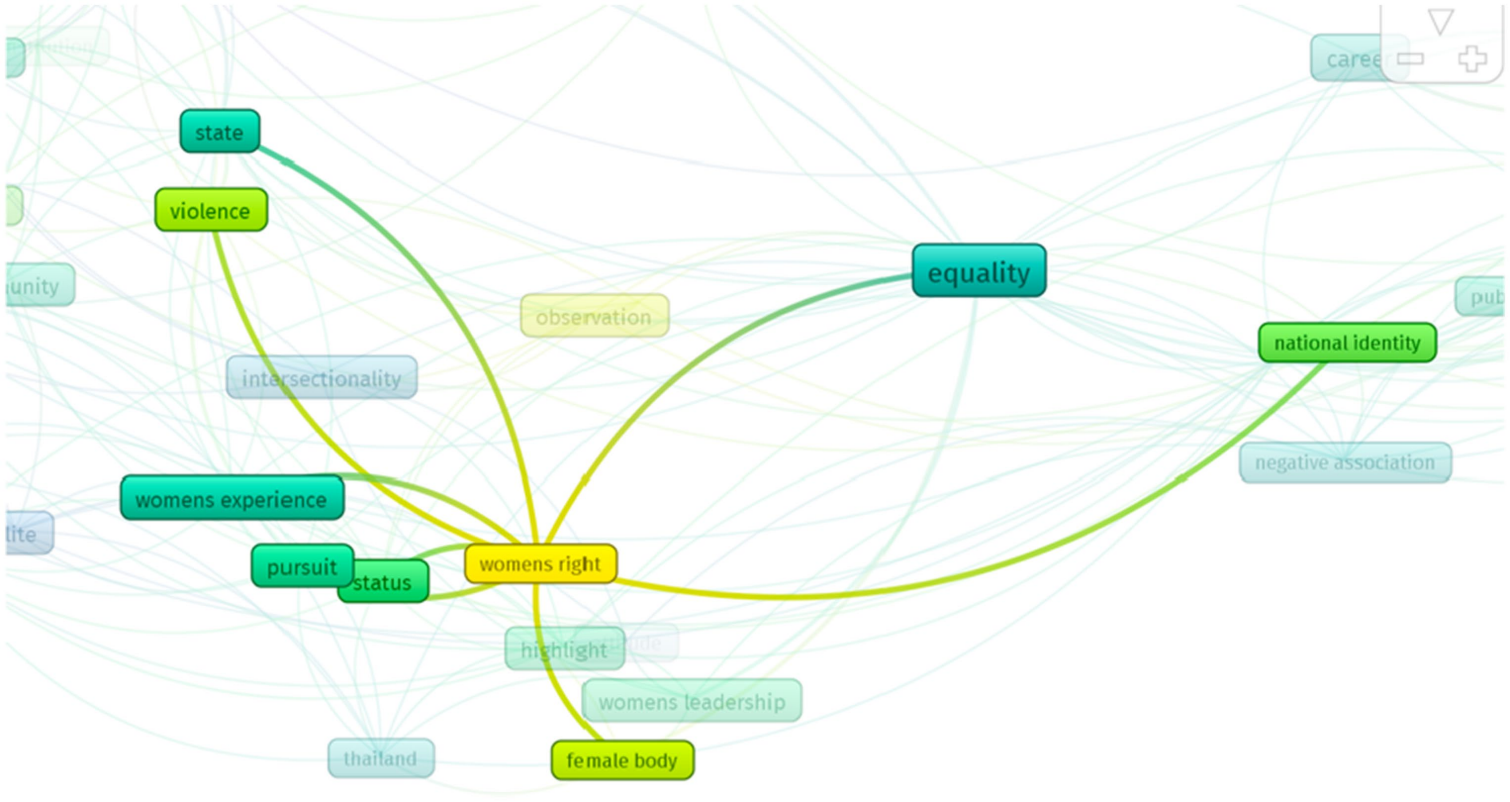
Women’s right term.

The lighter colors in Figure 6 indicate limited previous research on women’s rights. That term is related to *national identity*, *violence*, *female body*, *women’s experience*, *status*, and *state*. The visualization is in Figure 6.

**Figure 7:**
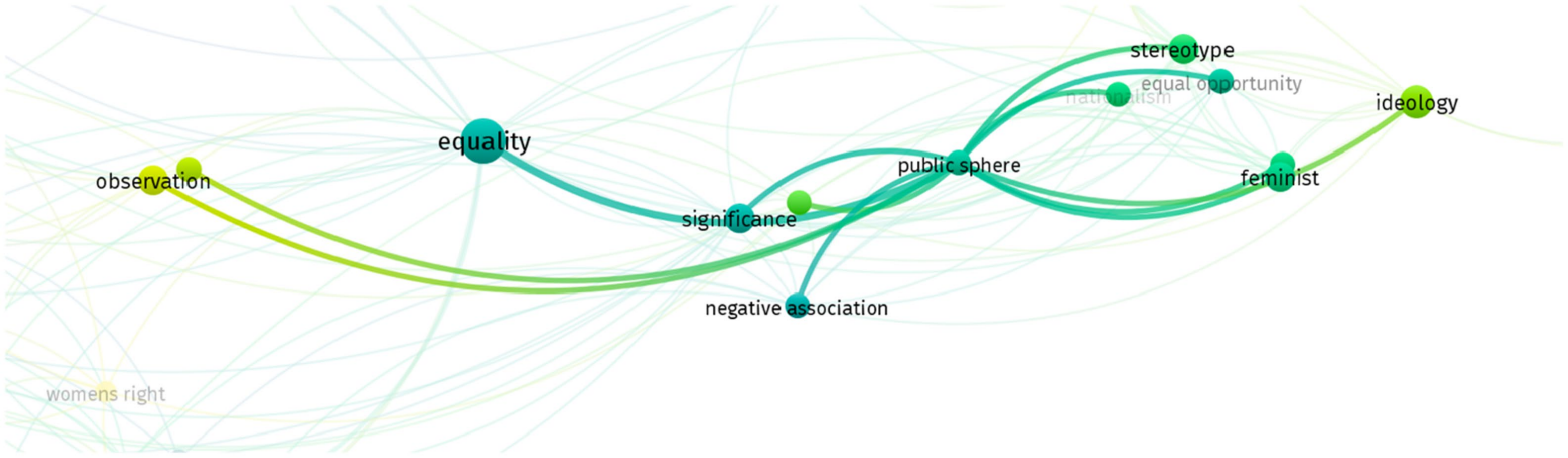
Public sphere term.

On the other hand, when highlighting the term public sphere, there is a significant relation to *equality*, *stereotype*, *feminist*, *negative association*, *ideology*, and *equal opportunity*. The visualization is in Figure 7.

The longstanding stigma on women from gender perspectives in the political field is not always true. In addition, this research shows that, in general, it has focused on the involvement of gender issues in public relations. This research challenges the patriarchal culture in public relations, wherein female practitioners have better management skills than male practitioners when given managerial tasks. The involvement of gender issues in leadership and sound management has profound implications for women’s leadership in social movements, particularly in the context of peace and security-based women’s empowerment. As Porter (2013) suggests, women’s leadership, often faced with unique challenges in addressing gender injustice and promoting inclusive peace, demonstrates a remarkable sensitivity to these issues. Panday (2020) findings further underscore the importance of gender issues in management and leadership, validating that when women have greater access to decision-making power, autonomy, and economic resources such as ownership of household assets, this not only enhances the well-being of individuals but also contributes significantly to the betterment of society.

Moreover, the indicators of women’s empowerment identified by Biswas and Kabir (2004), such as mobility, awareness of rights, participation in public protest, and contribution to family income, underscore the urgency of integrating gender issues in leadership and management. This integration is crucial for creating inclusive and productive work environments and for strengthening women’s role in leading broader and sustainable social change. It also highlights how women’s leadership in management can significantly enhance the effectiveness of advancing such issues in the context of social movements.

Amidst the efforts to improve women’s political participation, women’s political role has become divisive. Despite women (of Indonesian in this context) having a long history of political struggle, such as women figures who actively participated in the national independence movement, gender stereotypes and constraints continue to impede women’s participation in politics (Kriyantono et al., 2022; McGinley, 2008).

There has been a significant shift in the perception of women’s roles in public life, inspiring hope. This shift involves the dimensions of the quality of citizenship status, transcending the confines of legal status, as stated by Isin and Turner (2002), that

*“As a result, various struggles based upon identity and difference (whether sexual, “racial,”* “*ethnic”, diasporic, ecological, technological, or cosmopolitan) have found new ways of articulating their claims as claims to citizenship understood not simply as a legal status but as political and social recognition and economic redistribution.”*

It shows that knowledge in contemporary citizenship has advanced beyond legal formal status and has involved social and individual political recognition. Information about the development of the status of women in public spaces shows that women’s empowerment has become a topic in public spaces.

Research from various countries in Asia and Africa reveals an intriguing paradox. Despite women constituting over half of the population, they hold a disproportionately small number of political positions, whether appointed or elected at various government levels (Iwanaga, 2008). This paradox is not confined to these regions. In the West and Asia, women’s political participation has seen significant growth in various democratic Western countries, yet it remains lacking in Asian countries (Ahmad et al., 2019; Iwanaga, 2008). Ahmad et al. (2019) also highlight the gender separation in public political participation, where women face a disproportionate social status in a heavily patriarchal culture and political system.

Participation of women in social & economic aspects in Asia is influenced by various factors, e.g., social and cultural limitations, lack of government support, weak educational system, overregulation, and historical legacy. Nevertheless, research has shown that women are motivated and ready to join the workforce. Hence, many choose to go to urban areas for better opportunities (Kabbani and Arslan, 2019; Sarfaraz, 2018; Silova and Abdushukurova, 2009). Data from research by Women Research Institute in 2013 shows that the role of women’s organizations has improved, but there has not been much progress toward gender equality (WRI, 2013).

Research trends on gender and citizenship are extensive and complicated, reflecting the dynamic and complex nature of the problems. People from around the globe continue to debate whether the Constitution has given the same citizen rights to women. The context of historical constitutionalism and contemporary issues such as transgender rights and same-sex marriage influence this discussion.

Democratic citizenship is challenged by the fulfillment of gender and sexual rights in various countries, sparking debates on comprehending the relationship between the lack of explanation for sexual citizenship and the debates on the most recent gender and sex-based violence. In addition, there is a discourse on formulating a critical analysis of gender and sex-based violence toward a democratic understanding.

The significance of comparative and interdisciplinary study on religion, gender, and sexuality, similarly, remains a subject of debate. This debate emphasizes the importance of learning and comparing different identities and communities in various presently existing contexts and how such comparative studies provide similarities and disparities between different groups of people, not as significant differences but as differences in an ongoing process. This argument highlights why it is necessary to conduct research and discussion on gender and citizenship perpetually. It also underlines the need for an approach involving various disciplines and comparisons to understand this problem comprehensively.

The relationship between individuals and nations is not the only component of citizenship research in gender perspectives. It also comprises various social institutions, such as families, households, conventional systems, civil community organizations, economic institutions, etc. These institutions influence the lives and opportunities of both men and women. Women are often associated with men as daughters, sisters, or wives. Despite that, being a citizen should give them their rights to their own identity and rights (Cornwall and Rivas, 2015; Elder-Vass, 2017).

## Conclusion

Despite the accomplished advancement in addressing gender issues in social movements and gender equality empowerment, many problems persist. Deep-seated gender discrimination, negative stereotypes, and patriarchal culture may become significant obstacles for women to participate in politics. Therefore, it is important to provide a comprehensive theoretical review of this issue to properly understand the present challenges and future probabilities. Hence, by getting a deeper understanding of this issue, this article intends to offer broader and deeper perspectives on gender issues in politics, which may help to change social, economic, and political lives.

The research offers targeted recommendations for advancing gender studies in relation to women’s leadership and social movements. These include incorporating curricula that enhance awareness of gender issues in both formal and informal education settings. Moreover, there is a need for more comprehensive training for women leaders and those aspiring to leadership roles, aimed at fostering effective leadership skills and addressing the unique challenges that women encounter across different sectors. Additionally, establishing robust, supportive networks among women leaders and social activists is crucial for bolstering solidarity and broadening their impact within the wider movement for gender equality. In this study, potential new research paths include exploring effective strategies and training programs for cultivating women’s leadership skills, as well as identifying the factors that either facilitate or impede their advancement in leadership roles. By delving into this line of inquiry, we aim to deepen our understanding and develop more effective solutions for promoting gender equality throughout society. It’s important to note that this study was limited to utilizing the VOS Viewer visualization tool and conducting a Systematic Literature Review (SLR) with a focus on the Scopus search engine.

## Data Availability

The raw data supporting the conclusions of this article will be made available by the authors, without undue reservation.

## References

[ref1] Ahmad A. Mahmood Q. K. Saud M. Mas’udah S. (2019). Women in democracy: the political participation of women. Masyarakat, Kebudayaan Dan Politik *32* :114. doi: 10.20473/mkp.V32I22019.114-122

[ref2] Akchurin M. Lee C.-S. (2013). Pathways to empowerment: repertoires of women’s activism and gender earnings equality. Am. Sociol. Rev. *78* , 679–701. doi: 10.1177/0003122413494759

[ref3] Arifin S. (2020). Kesetaraan gender dan pertumbuhan ekonomi di Indonesia. Kajian *23* , 27–42. Available at: https://jurnal.dpr.go.id/index.php/kajian/article/view/1872

[ref4] Audina D. J. (2022). Kesetaraan gender dalam perspektif hak asasi manusia. Nomos: Jurnal Penelitian Ilmu Hukum *2* , 148–154. doi: 10.56393/nomos.v1i6.602

[ref5] Aziz A. Shams M. Khan K. S. (2011). Participatory action research as the approach for women’s empowerment. Action Res. *9* , 303–323. doi: 10.1177/1476750310396952

[ref6] Biswas T. K. Kabir M. (2004). Measuring women’s empowerment: indicators and measurement techniques. Social Change *34* , 64–77. doi: 10.1177/004908570403400305

[ref7] Blanton S. L. Peksen D. Blanton R. (2023). The impact of peacekeeping missions on women’s empowerment. Polit. Res. Q. *76* , 1872–1887. doi: 10.1177/10659129231181594

[ref8] Cameron L. (2023). Gender equality and development: Indonesia in a global context. Bull. Indones. Econ. Stud. *59* , 179–207. doi: 10.1080/00074918.2023.2229476

[ref9] Cornwall A. Rivas A.-M. (2015). From ‘gender equality’ and ‘women’s empowerment’ to global justice: reclaiming a transformative agenda for gender and development. Third World Q. *36* , 396–415. doi: 10.1080/01436597.2015.1013341

[ref10] Davies S. E. (2018). Gender empowerment in the health aid sector: locating best practice in the Australian context. Aust. J. Int. Aff. *72* , 520–534. doi: 10.1080/10357718.2018.1534938

[ref11] Elder-Vass D. (2017). Book review: religion, gender and citizenship: women of faith, gender equality and feminism. Sociol. Rev. *65* , 429–431. doi: 10.1177/0038026117701343

[ref12] Evans C. A. Mayo L. M. Quijada M. A. (2018). Women’s empowerment and nonprofit sector development. Nonprofit and voluntary sector. Nonprofit Volunt. Sect. Q. *47* , 856–871. doi: 10.1177/0899764018764331

[ref13] Fielding-Miller R. Hatcher A. M. Wagman J. Swendeman D. Upadhyay U. D. (2020). Gender, justice and empowerment: creating the world we want to see. Cult. Health Sex. *22* , 1–12. doi: 10.1080/13691058.2020.1736843, PMID: 32723225 PMC8155812

[ref14] Foucault M. (1977). Discipline and punish: The birth of the prison. New York: Vintage Books.

[ref15] Holmarsdottir H. B. Ekne I. B. M. Augestad H. L. (2011). The dialectic between global gender goals and local empowerment: girls’ education in southern Sudan and South Africa. Res. Comp. Int. Educ. *6* , 14–26. doi: 10.2304/rcie.2011.6.1.14

[ref16] Isin E. Turner B. (2002). Handbook of citizenship studies. Thousand Oaks, CA: Sage.

[ref17] Iwanaga K. (2008). Women’s political participation and representation in Asia: Obstacles and challenges. Copenhagen: NIAS Press.

[ref18] Kabbani N. S. Arslan A. S. (2019). Investing in rural youth in the near east, North Africa, Europe and Central Asia. *Rural Development Report*.

[ref19] Kent C. (2016). “Surveying and being surveyed: gender aspects in John Berger’s ways of seeing” in On John Berger. eds. Ralf H. David M.. (BRILL), 211–232.

[ref20] Kirton G. (2021). Union framing of gender equality and the elusive potential of equality bargaining in a difficult climate. J. Ind. Relat. *63* , 591–613. doi: 10.1177/00221856211003604

[ref21] Klasen S. Lamanna F. (2009). The impact of gender inequality in education and employment on economic growth: new evidence for a panel of countries. Fem. Econ. *15* , 91–132. doi: 10.1080/13545700902893106

[ref22] Kriyantono R. Ida R. Tawakkal G. T. I. Safitri R. (2022). Not just about representative: when democracy needs females and their competency to run Indonesian government public relations to management level. Heliyon *8* :e08714. doi: 10.1016/j.heliyon.2022.e08714, PMID: 35036606 PMC8752903

[ref23] McGinley A. C. (2008). Hillary Clinton, Sarah Palin, and Michelle Obama: performing gender, race, and class on the campaign trail. Denv. UL Rev. *86* :709. Available at: https://scholars.law.unlv.edu/facpub/171/

[ref24] Mitander T. (2024). Undoing the regional demos? Gender equality and economic growth in regional development. NORA Nord. J. Fem. Gend. Res. 32, 49–61. doi: 10.1080/08038740.2023.2201474

[ref25] Mulvey L. (1975). Visual pleasure and narrative cinema. Screen *16* , 6–18. doi: 10.1093/screen/16.3.6

[ref26] Orfan S. N. Samady S. (2023). Students’ perceptions of gender equality: a case study of a conflict-stricken country. Cogent Soc. Sci. 9, 1–19. doi: 10.1080/23311886.2023.2225819

[ref27] Page M. J. McKenzie J. E. Bossuyt P. M. Boutron I. Hoffmann T. C. Mulrow C. D. . (2021). The PRISMA 2020 statement: an updated guideline for reporting systematic reviews. BMJ. 372, 1–19. doi: 10.1136/bmj.n71, PMID: 33782057 PMC8005924

[ref28] Panday P. (2020). Women’s empowerment and the well-being of children in Nepal. J. Dev. Soc. *36* , 129–154. doi: 10.1177/0169796X20916048

[ref29] Patel T. Romani L. Oberoi P. Ramasamy C. (2023). Gender role encapsulation as resistance to patriarchy: women politicians’ work and gender equality in India. Organization *30* , 307–325. doi: 10.1177/1350508421995764

[ref30] PCAR . (2013). *Sex, gender, gender expression, and gender identity biological sex*. Available at: www.uta.edu/english/timothyr/Fausto-Sterling (Accessed March 18, 2024).

[ref31] Pervaiz Z. Akram S. Ahmad Jan S. Chaudhary A. R. (2023). Is gender equality conducive to economic growth of developing countries? Cogent Soc. Sci. 9, 1–16. doi: 10.1080/23311886.2023.2243713

[ref32] Porter E. (2013). Rethinking women’s empowerment. J. Peacebuilding Dev. *8* , 1–14. doi: 10.1080/15423166.2013.785657

[ref33] Quek K. M.-T. Knudson-Martin C. (2008). Reshaping marital power: how dual-career newlywed couples create equality in Singapore. J. Soc. Pers. Relat. *25* , 511–532. doi: 10.1177/0265407508090871

[ref34] Rahman M. W. Haque A. B. M. M. Zaman T. Palash M. S. Nahiduzzaman M. Nazia T. (2024). Women empowerment status in the coastal fishing communities of Bangladesh. SAGE Open. 14, 1–16. doi: 10.1177/21582440241250114 PMC10990856 38576559

[ref35] Sarfaraz H. (2018). Women labor force participation in Pakistan: a survey. Int. J. Women Empower. 4, 8–13. doi: 10.29052/2413-4252.V4.I1.2018.8-13

[ref36] Sari G. R. Ismail E. (2021). Polemik pengarusutamaan kesetaraan gender di Indonesia. Jurnal Penelitian Ilmu Ushuluddin *1* , 51–58. doi: 10.15575/jpiu.12205

[ref37] Scambor E. Bergmann N. Wojnicka K. Belghiti-Mahut S. Hearn J. Holter Ø. G. . (2014). Men and gender equality. Men Masculinities *17* , 552–577. doi: 10.1177/1097184X14558239

[ref38] Silova I. Abdushukurova T. (2009). Global norms and local politics: uses and abuses of education gender quotas in Tajikistan. Glob. Soc. Educ. *7* , 357–376. doi: 10.1080/14767720903166376

[ref39] Stowers K. Hancock G. M. Neigel A. Cha J. Chong I. Durso F. T. . (2019). HeForShe in HFE: strategies for enhancing equality in leadership for all allies. Proc. Hum. Factors Ergon. Soc. Annu. Meet. *63* , 622–624. doi: 10.1177/1071181319631382

[ref40] Suhada D. N. (2021). Feminisme dalam dinamika perjuangan gender di Indonesia. Indones. J. Sociol. Educ. Dev. *3* , 15–27. doi: 10.52483/ijsed.v3i1.42

[ref5001] VOSViewer . (2024). VOSviewer version 1.6.19. [Application]. Available at: https://www.vosviewer.com/downloads/VOSviewer_1.6.20_mac.zip

[ref41] Wang X. Chui W. H. Wang Y. (2022). Perception of gender equality matters: targets’ responses to workplace sexual harassment in Chinese metropolises. J. Interpers. Violence *37* :NP11933–NP11963. doi: 10.1177/0886260521997452, PMID: 33648365

[ref42] WRI . (2013). Gerakan perempuan bagian gerakan demokrasi di Indonesia. *Jurnal Afirmasi*.

